# Autochthonous *Thelazia callipaeda* Infection in Dog, New York, USA, 2020

**DOI:** 10.3201/eid2707.210019

**Published:** 2021-07

**Authors:** A.B. Schwartz, Manigandan Lejeune, Guilherme G. Verocai, Rebecca Young, Paul H. Schwartz

**Affiliations:** Marist College, Poughkeepsie, New York, USA (A.B. Schwartz);; Animal College of Veterinary Medicine at Cornell University, Ithaca, New York, USA (M. Lejeune, R. Young);; Texas A&M University College of Veterinary Medicine and Biological Sciences, College Station, Texas, USA (G.G. Verocai);; Center for Veterinary Care Millbrook, PC, Millbrook, New York, USA (P.H. Schwartz)

**Keywords:** *Thelazia callipaeda*, *Thelazia*, vector-borne infections, emerging diseases, zoonotic diseases, helminth, parasite, oriental eye worm, eye worm, dog, canine, zoonoses, United States

## Abstract

We report a case of autochthonous infection of the eye worm *Thelazia callipaeda* in a dog in the northeastern United States. Integrated morphologic identification and molecular diagnosis confirmed the species. Phylogenetic analysis suggested introduction from Europe. The zoonotic potential of this parasite warrants broader surveillance and increased awareness among physicians and veterinarians.

Thelaziasis in dogs can be caused by 2 nematodes of the genus *Thelazia* (Nematoda: Spirurida): *T. callipaeda* and *T. californiensis* (*1*). The oriental eye worm (*T. callipaeda*) is a helminth that infects a variety of domestic and wild carnivores, lagomorphs, rodents, and primates (including humans) across Eurasia (*2*,*3*). In Europe, the *T. callipaeda* eye worm is an emergent vectorborne helminth that has spread steadily across all countries over the past 3 decades (*2*). The California eye worm (*T. californiensis*) has been reportedly found in wild and domestic carnivores, ungulates, lagomorphs, and humans; its range is limited to the western United States (*4*). Zoonotic infection of humans with a third species of eye worm (*T. gulosa*), which infects cattle, has recently been reported in the western United States (*5*). These 3 species of *Thelazia* eye worm with zoonotic potential are morphologically and biologically distinct (*1*,*5*,*6*).

*Thelazia* nematodes are found in the conjunctival recesses of the eye (*1*). Secretophagous dipteran intermediate hosts (*1*,*7*) ingest first-stage larvae (L1) while feeding from the definitive host’s eyes. After metamorphosis, infective third-stage larvae (L3) are passed via the labelum onto the conjunctiva of another suitable host. L3 develop into adults that migrate to the conjunctival recess, lacrimal ducts, or both, resulting in conjunctivitis, ocular discharge, and blepharospasm. Female worms release more L1, seeding ocular secretions of the host, and conclude the life cycle (*1*,*2*). Intermediate hosts for *Thelazia* nematodes are dipteran flies of the genera *Phortica* for *T. callipaeda, Fannia* for *T. californiensis*, and *Musca* for *T. gulosa* (*1*,*5*,*7*,*8*). *P. variegata* fruit flies are widely distributed across Eurasia and have been found in multiple areas in the eastern United States (*9*). In North America, they have been experimentally proven to be competent vectors for *T. callipaeda* worms (*10*), supporting the potential occurrence of *T. callipaeda* infection in the United States (*2*). We report *T. callipaeda* eye worm infection detected in a dog in the Western Hemisphere in November 2020.

## The Case

The patient was a 7.5-year-old Labrador retriever, with no relevant medical history. The dog routinely received heartworm preventive (Heartgard; Boehringer Ingelheim Pharmaceuticals, Inc., https://www.boehringer-ingelheim.com) and flea and tick preventive (NexGard [Boehringer Ingelheim Pharmaceuticals, Inc.] and Vectra [Ceva Animal Health, https://www.ceva.us]) in accordance with recommended dosing and had never traveled beyond Dutchess County, New York, USA. The dog was taken to a veterinarian because of a 3-week history of unilateral epiphora and blepharospasm. Treatment with an ophthalmic preparation (neomycin, polymyxin B, and dexamethasone) produced no demonstrable response. Subsequent nasolacrimal duct flushing with 0.3% gentamicin sulfate and 0.2% dexamethasone in physiological saline solution, followed by 100 μg/mL ivermectin in physiological saline solution, led to recovery of 12 nematodes. After systemic ivermectin administration, no recurrence has been noted. 

**Figure 1:**
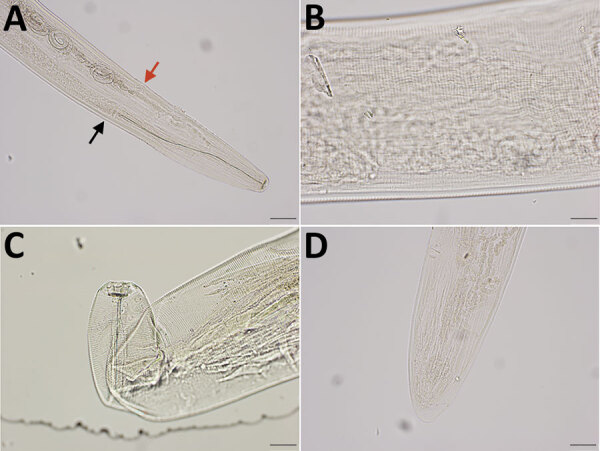
Integrated diagnostic approach for confirming *Thelazia callipaeda* nematodes: morphologic identification. Specimens were cleared in lactophenol before examination under an Olympus compound microscope (BX53) (https://www.olympus-lifescience.com). Images were taken with an Olympus DP73 camera, and morphometry was performed by using Olympus cellSens software. A) Cephalic end of a female worm. Black arrow indicates esophageal intestinal junction; red arrow indicates vulval opening. Original magnification ×100. B) Transverse striations (150–190/mm) in the cuticle of midbody region of a female worm. Original magnification ×200. C) Buccal cavity of a female worm, wider than deep. Note tightly spaced cuticular striations in the cephalic end. Original magnification ×200. D) Caudal end of female worm with protruding phasmids in the tip. The tail was not protruding unilaterally. Original magnification ×100.

Four nematodes (3 female, 1 male) were morphologically identified as *T. callipaeda* eye worms on the basis of the cuticular transverse striations (CTS) pattern and vulva position (*1*,*6*). The 3 female worms were 12.7–13.9 mm long and 314–360 μm wide. The vulval opening was anterior to the esophageal intestinal junction, and in 1 specimen it was 610.86 μm from the cephalic end. The midbodies contained 150–190 CTS/mm, and the cephalic/caudal region contained 220–240 CTS/mm. The buccal capsule was wider than it was deep. Two protruding phasmids were visible at the tip of the tail, which did not taper unilaterally (Figure 1). The male worm was 8.9 mm long, and its width was not measured. The cephalic region contained 310 CTS/mm, and the midbody/caudal region contained 170 CTS/mm. The small spicules measured 147.73 μm; the large spicules, 1721.90 μm.

**Figure 2:**
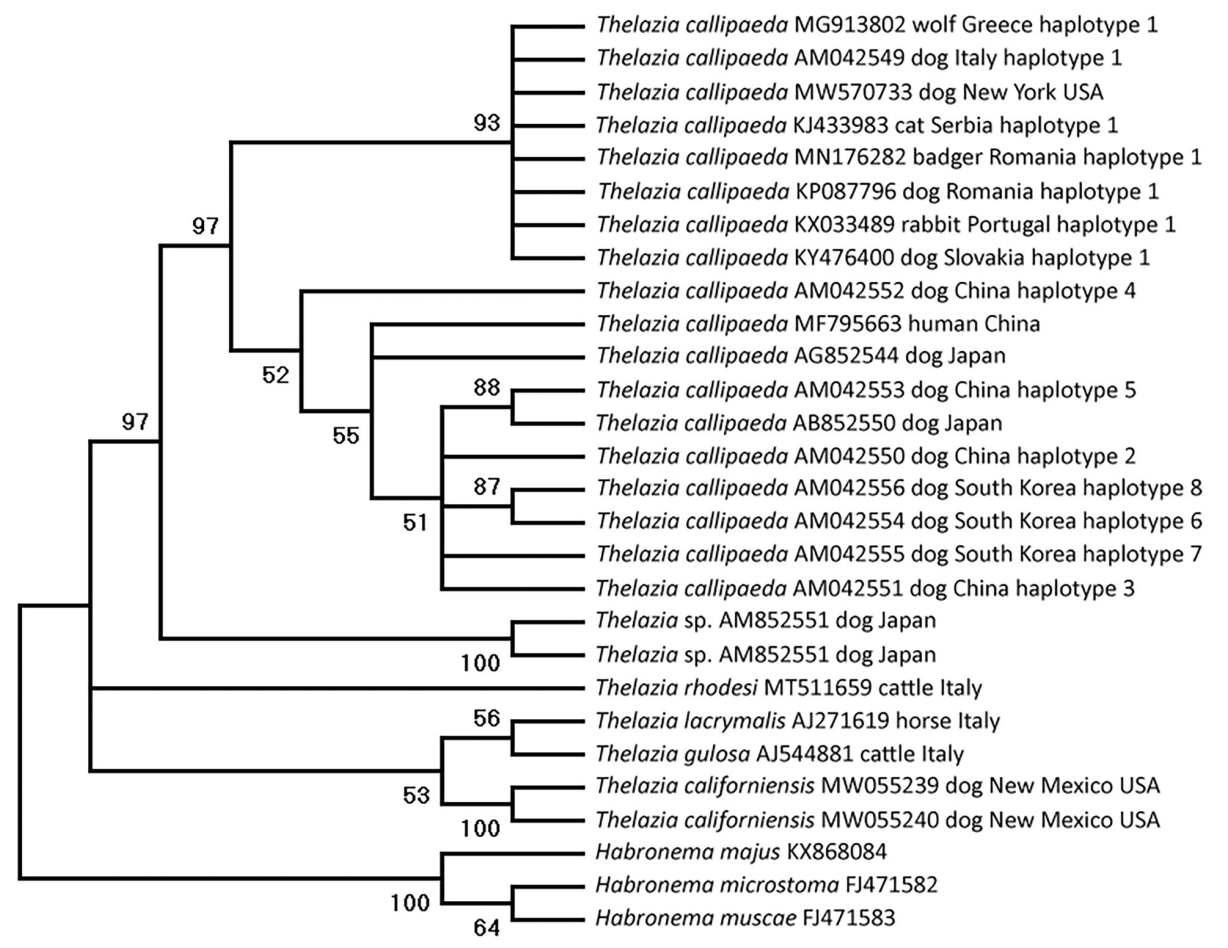
Phylogenetic relationship based on cytochrome oxidase c subunit 1 gene of *Thalazia callipaeda* nematode isolate from a dog in Dutchess County, New York, USA, 2020 (GenBank accession no. MW570733), and other related *Thelazia* species available in GenBank (accession numbers shown). Analysis was performed by using MEGAX (http://www.megasoftware.net) and the maximum-likelihood method, 1,000 bootstrap replicates; nodes with <50% support were condensed.

We subjected a female worm to DNA extraction and multilocus PCR (18S rRNA, 12S rRNA, and cytochrome oxidase c subunit 1 [*cox1*] gene markers) by using assays described previously (*4*,*11*), followed by sequencing. The partial sequences generated for 18S rRNA matched 99.7%, 12S rRNA 99.4%, and *cox1* genes 93.3%–100% of the corresponding genes of *T. callipaeda* worms in GenBank. We deposited the generated sequences in GenBank (accession no. MW570771 for 18S rRNA, MW575766 for 12S rRNA, and MW570733 for *cox1*). Molecular data unequivocally confirmed the parasite as *T. callipaeda*. Phylogenetic analysis of the *cox1* gene showed that the *T. callipaeda* eye worm found in North America belongs to the haplotype-1 prevalent in Europe (100% maximum identity), suggesting a possible source of introduction (Figure 2).

## Conclusions

The discovery of an autochthonous case of *T. callipaeda* eye worm infection in the United States suggests its introduction and establishment on a continent where natural infection has not been documented. The report on the distribution of *P. variegata* fruit flies in the eastern United States and their competence for *T. callipaeda* eye worms has raised concern for eye worm infections in animals and humans in this region (*10*). Our finding is in line with previous predictions. Active surveillance of all susceptible hosts, coupled with ecologic niche modeling as conducted in Europe, can help gauge the extent of *T. callipaeda* eye worm spread in North America (*7*). Our findings should bring awareness about this invasive, zoonotic parasite to veterinary and medical ophthalmologists in the Americas. To curtail the potential spread in the United States, consideration should be given to US Department of Agriculture–imposed requirements for implementing broad and accurate parasite diagnostic methods and prophylactic anthelmintic treatment to mitigate the introduction of exotic parasites before relevant species are imported. The One Health model of cooperation among veterinarians, wildlife biologists, and physicians is vital for assessing the current distribution and mitigating the spread of this multihost parasite with zoonotic potential. As this case shows, emergence of *T. callipaeda* parasites requires increased awareness by both human medical and veterinary professionals.
